# Anxiety and depression in patients who undergo a cerebrovascular procedure

**DOI:** 10.1186/s12883-020-01674-8

**Published:** 2020-04-07

**Authors:** Lauren Lombardo, Richard Shaw, Kathleen Sayles, Dorothea Altschul

**Affiliations:** 1 The Valley Hospital, Ridgewood, NJ USA; 2 Columbia College of Physicians and Surgeons, New York, NY USA

**Keywords:** Cerebrovascular disease, Outcomes research, Quality of life, Depression, Neurointerventional

## Abstract

**Background:**

Observe the relationship of anxiety and depression on quality of life outcomes after open and endovascular cerebrovascular procedures.

**Methods:**

We retrospectively analyzed 349 patients who underwent a procedure for aneurysm, arteriovenous malformation, intraparenchymal hemorrhage, carotid stenosis, acute stroke, and conventional catheter angiogram over three years at a community hospital. We correlated pre-procedural anxiety and depression with Global Physical Health, Global Mental Health, and Modified Rankin Scale scores. We performed univariate and multivariate linear and logistic regression analyses adjusting for past medical history and sociodemographic factors.

**Results:**

Anxiety or depression occurred in 18 % of patients. Patients with anxiety or depression were more likely to be female (81% vs 60.8%; *p* = 0.002) and younger (54 vs. 59 years old; *p* = 0.025). The groups did not differ in type or urgency of procedure, smoking or history of diabetes. Patients with anxiety or depression reported lower mental health scores at 30 days (45.1 vs 48.2; *p* = 0.002) post-procedure. In multivariate analyses, anxious or depressed patients had worse mental health scores at 30 days (t = − 2.893; *p* = 0.008) than those who did not have a history of anxiety or depression. There was no difference between groups in length of stay, mortality, physical health t-scores, functionality scores, or six month quality of life outcomes.

**Conclusions:**

Patients undergoing cerebrovascular procedures who self-reported anxiety or depression showed a significant difference in mental health outcomes at 30 days, but six month mental health and other medical and functional outcomes measures were similar to patients without these diagnoses.

## Background

Mental health disorders are common in the United States but they are not usually the focus of a conversation about a patient’s medical history before they undergo a cerebrovascular procedure [7]. Understanding the impact of a cerebrovascular procedure on an individual already experiencing anxiety or depression (Anx/Dep) may help provide more realistic expectations for quality of life (QOL) outcomes for this group, which is especially important if they will fair differently than similar patients without Anx/Dep. This knowledge could give patients who experience Anx/Dep the opportunity to ensure that their chosen mental health services are available to them before and after their procedure to combat a possibly lower QOL outcome than those who do not experience Anx/Dep.

We hypothesized that patients who reported Anx/Dep as part of their medical record would have worse physical and mental health outcomes at 30 days and six months after their cerebrovascular procedure than those who did not. To our knowledge, no prior study has analyzed this relationship despite the prevalence of Anx/Dep in the population. The purpose of this retrospective study is to describe the effect of having a cerebrovascular procedure on QOL outcomes for those who experience Anx/Dep using the ten item Patient Reported Outcomes Measurement Information System Global Health Scale (GHS) and simplified Modified Rankin Scale (mRS). We chose to use mRS because it is a well-known valid and reliable scale of functional disability.

## Materials and methods

### Sample

The study was conducted retrospectively at The Valley Hospital, a community hospital in Ridgewood, NJ, for a three-year period from July 1, 2014 to June 30, 2017. The baseline cohort included 424 consecutive neurological procedures. In order to be included in the study sample, patients had to be eighteen years of age or older on the date of their procedure and have 30 day follow up information available. Accounting for these inclusion criteria, the final analytical sample consisted of 349 procedures. Qualifying procedures included cerebrovascular procedures for intracranial embolization or craniotomy for cerebral aneurysms or arteriovenous malformations or intraparenchymal hemorrhages (IPH) (*n* = 78), carotid endarterectomy or stenting (*n* = 6), acute stroke intervention (*n* = 16), and conventional cerebral catheter angiogram (*n* = 249). Both elective (*n* = 315) and urgent (*n* = 34) procedures were included.

Data were collected from the patients’ electronic medical record, including any report of a past or present experience with anxiety, depression, or both. Of the 349 patients with cerebrovascular procedures, there were 63 patients (18.1%) who self-reported as part of their past medical history a prior or current diagnosis of anxiety (*n* = 27), depression (*n* = 18), or both (n = 18). Due to the small sample size when broken down into these categories, we combined them into one group for our analysis.

Baseline variables included sociodemographic information and past medical history as shown in Table 1. This included factors such as a history of smoking, alcohol consumption, and migraine. Medication records were not considered for the purpose of this analysis.

**Table 1:** Baseline Sociodemographic Information

Groups	With Anx/Dep (*N* = 63)	Without Anx/Dep (*N* = 286)	*p* value
Mean age	54.3 ± 15	58.7 ± 14	0.025
Female	51 (81.0%)	174 (60.8%)	0.002
Ethnicity			0.064
Caucasian	56 (88.9%)	225 (78.7%)	
Non-Caucasian	7 (11.1%)	61 (21.3%)	
Smoking			0.839
Former/Never	54 (85.7%)	248 (86.7%)	
Every day/Occasional	9 (14.3%)	38 (13.3%)	
Alcohol			0.254
Never/Rare	37 (58.7%)	187 (65.4%)	
Occasional/Social	21 (33.4%)	90 (31.5%)	
Heavy/Abuse/Hx of Abuse	5 (7.9%)	9 (3.1%)	
Diabetes	5 (7.9%)	33 (11.5%)	0.507
Hypertension	33 (52.4%)	154 (53.8%)	0.833
Type of Procedure (total = *n*)			0.837
Cerebral Angiogram (*n* = 249)	45 (71.4%)	204 (71.3%)	
Intracranial/Craniotomy (*n* = 78)	15 (23.8%)	63 (22.1%)	
Endarterectomy/stent (*n* = 6)	1 (1.6%)	5 (1.7%)	
Stroke (*n* = 16)	2 (3.2%)	14 (4.9%)	

This study was approved by the Western Institutional Review Board and was exempt from obtaining informed consent due to being a retrospective chart review. The data supporting the conclusions of this manuscript will be made available by the authors, without undue reservation, to any qualified researcher.

### Outcomes variables

As part of standard clinical protocol, patients completed the GHS and mRS assessments either by hand or interview at the time of their procedure as well as 30 days (target 28–45 days, *n* = 349) and six months (target ±2 weeks) post procedure (*n* = 326). Patient responses to each of the ten GHS items were scored on a scale from 1 to 5 (1 = poor, 2 = fair, 3 = good, 4 = very good, 5 = excellent) [3].

These raw scores converted to a Global Mental Health (GMH) score and a Global Physical Health (GPH) score, each using four GHS items that pertained to mental or physical health. The t-scores ranged from 21.2–67.7 for GMH scores and 16.2–67.7 for GPH scores [3]. A higher t-score indicated a better outcome. The average t-score for the general population of the United States is 50. Respondents who fall within one standard deviation of the mean will score a GMH t-score in the range of 41.1–59 or a GPH t-score within the range of 42.3–57.7 [3]. The questionnaires provided to the subjects were in English. We excluded anyone who did not complete a 30 day questionnaire. Patients who were excluded were older and had a higher mRS score and lower GPH score at baseline, but they were similar in their pre-morbid reports on Anx/Dep when compared to our final analytical sample. GHS scores were not used to determine if the patient had Anx/Dep.

The mRS is a measure of functionality commonly used to evaluate patients after a stroke or cerebrovascular accident [1]. The mRS is scored on a scale from 0 to 6 where 0 is no symptoms, 5 is confined to bed, and 6 is death. A simplified mRS questionnaire was used to improve reliability [1]. For the purpose of this study, patients were split into groups where an mRS of 0–2 was considered an outcome with little to no disability and an mRS of 3 to 5 meant moderate to severe disability. Twelve patients were found to be deceased at their 30 day follow up (0.02%). They were excluded from this analysis because they did not have 30 day follow up information.

Length of hospital stay and days spent in the intensive care unit (ICU) were recorded from the medical record and are presented in Table 2. We also looked at hospital complications that were recorded in the medical record such as requiring re-intubation, urinary tract infections, and hyper- or hypoglycemia.

**Table 2:** Results of univariate and multivariate analyses to determine association of Anx/Dep status with outcome*

	Analyses	Univariate		Multivariate	Anx/Dep vs. None	
	Anx/Dep	None	p value	β (95% CI)	t value	p value
Length of Stay (days)	3.7 ± 6	4.2 ± 6	0.619	.017 (−1.39–1.94)	0.316	0.752
ICU Days	1.7 ± 4	2.0 ± 4	0.515	.004 (− 1.04–1.14)	0.083	0.934
30 Day Follow Up						
GHS Mental	45.1 ± 7	48.2 ± 7	0.002	−.145 (−4.8 - -.8)	−2.893	0.008
GHS Physical	44.4 ± 7	46.5 ± 8	0.063	−.099 (− 4.3–0.19)	−1.799	0.073
6 Month Follow Up						
GHS Mental	47.5 ± 7	49.1 ± 7	0.098	−0.085 (−3.7–.47)	−1.523	0.129
GHS Physical	45.6 ± 6	46.8 ± 8	0.283	−.055 (−3.2–1.1)	−0.985	0.325

* These multivariate analyses included age, gender, type and urgency of procedure, smoking, diabetes, and Anx/Dep. Logistic regression multivariate analyses of mRS at 30 days demonstrated non-significant adjusted odds ratios of 3.8 (95% CI 0.88–16.9; *p* = 0.071) and 2.7 (95%CI 0.76–9.52; *p* = 0.126) at 6 months for Anx/Dep vs. None

### Statistical analysis

Univariate variables are presented as percentages and were analyzed using the Person Chi-Square and t-test. Multivariate logistic and linear regression analyses were used to evaluate dichotomous outcomes and all analyses were adjusted for age, gender, type and urgency of procedure, smoking, diabetes, and hypertension. In this sample specifically, type and urgency of procedure were not found to be statistically significant confounders. A *p*-value of p less than 0.05 is statistically significant. IBM/SPSS statistical package (Version 19.0) performed all statistical analyses.

## Results

In our total sample, the average age was 56.5 years old and 64.5% were women. Patients were 85.5% Caucasian. Occasional or everyday smokers made up 13.5% of our sample. Ninety one percent of the sample was comprised of elective procedures. Further details regarding the composition of the sample including, type of procedure, sociodemographic factors, and past medical history are described in Table 1.

We found that 18.1% of our sample reported Anx/Dep. Patients with Anx/Dep were significantly younger and more likely to be female than patients who did not have a past medical history indicating Anx/Dep. There were no significant differences between those with or without Anx/Dep in type or urgency of procedure, diagnosis (Table 4), smoking or alcohol consumption, or history of diabetes. Ethnicity did not have an effect on outcomes but our sample of non-Caucasian patients was too small to draw a firm conclusion.

Additionally, there was no significant difference between Anx/Dep and non-Anx/Dep patients in ICU days (1.7 ± 4 vs 2.0 ± 4; *p* = 0.515) or hospital length of stay (3.7 ± 6 vs. 4.2 ± 6; *p* = 0.619), also shown in Table 2. There was no significant difference between Anx/Dep and non-Anx/Dep in discharge disposition to home (82.5% vs. 78.7%; *p* = 0.316) or to other intermediate care facilities. In-hospital complications were not significantly different between the two groups. There was no significant difference between Anx/Dep and non-Anx/Dep patients on mRS scores 3 to 5 at 30 days (6.3% vs. 3.2%; *p* = 0.264) or at 6 months post procedure (8.6% vs. 5.2%; *p* = 0.388).

When using a multivariate analysis controlling for baseline demographics and clinical factors, patients with Anx/Dep had a lower GMH score at their 30 day follow up that was of statistical significance (*p* = 0.002). Other medical and functional outcome measures such as diabetes, alcohol use, and smoking, as well as six month assessment of GPH, GMH, and mRS were similar to patients without these diagnoses (Table 2). GPH, GMH, mRS, and Anx/Dep status were broken down by procedure type in Table 3 and by diagnosis in Table 4.

**Table 3:** Results Based on Procedure Type

	Mental Health T-Score at 30-Days	Physical Health T Score at 30-Days	MRS 0–2 at 30 Days	MRS 3–5 at 30 days	Anxiety/Depression
Diagnostic Angiogram	47.9 ± 8	46.5 ± 8	98.0%	2.0%	18.1%
Open Vascular Procedure	46.9 ± 7	46.4 ± 7	93.9%	6.1%	30.3%
Endovascular procedure	47.4 ± 8	44.7 ± 7	95.9%	4.1%	10.2%
Mechanical Thrombectomy	46.0 ± 7	44.2 ± 8	77.8%*	22.2%	16.7%

*p = 0.03

**Table 4:** Results Based on Diagnosis

	Mental Health T-Score at 30-Days	Physical Health T- Score at 30-Days	MRS 0–2 at 30 Days	MRS 3–5 at 30 Days	Anxiety/Depression
Aneurysm	46.5 ± 8	44.9 ± 7	93.6%	6.4%	23.4%
Arteriovenous Malformation (AVM)	47.1 ± 6	47.2 ± 5	100.0%	0.0%	0.0%
Carotid Stenosis	48.4 ± 6	50.8 ± 8	100.0%	0.0%	16.7%
Ischemic Stroke	46.4 ± 7	44.7 ± 8	81.3%	18.8%	12.5%
Intraparenchymal Hemorrhage	42.3 ± 15	40.2 ± 14	50.0%	50.0%	50.0%
Other^a^	47.9 ± 7	46.1 ± 8	97.8%	2.2%	17.5%

^a^Any other cerebrovascular diagnosis that was not classified as Aneurysm, AVM, Carotid Stenosis, Ischemic Stroke, or Intraparenchymal Hemorrhage but led to a cerebrovascular procedure

There was no change in the results if acute stroke patients (*n* = 16) were removed despite stroke patients having a significantly lower mRS at 30 days. Results were also the same after removing patients who reported having migraines with or without aura from the analysis (*n* = 36). There was one mortality in each group between 30 day and six month follow ups, which was not statistically significant (*p* = 0.329).

## Discussion

In this retrospective study of 349 patients undergoing a procedure for cerebrovascular disease, we found that anxiety and depression (Anx/Dep) are associated with worse Global Mental Health (GMH) scores at 30 days post procedure without any differences in Global Physical Health (GPH) or functional capacity (mRS) scores. This study is novel due to its focus on cerebrovascular procedures and mental health. In addition, our study benefits from our collection of detailed hospital complications and baseline medical comorbidity data.

Depression is experienced by 33 % of patients after a stroke [8]. Our large sample size and collection of many confounding variables allowed us to confirm that our results were unaffected even after removing stroke patients and those with migraines from our analysis.

To our knowledge, there is one other study that focused on cerebral aneurysms and Anx/Dep. This study analyzed the mental health status of 166 patients who presented to a neurosurgery clinic with a cerebral aneurysm [5]. They found that 8 % of their study population had depression and 17 % had anxiety [5]. Another study showed that mRS were significantly higher in depressed post-stroke patients than in non-depressed patients [4]. While the prevalence of Anx/Dep in patients in these studies and ours is similar, the studies are not entirely comparable. Our study focuses on quality of life outcomes (QOL) for patients with and without Anx/Dep who had a cerebrovascular procedure. Our patient sample is larger and includes a wider variety of cerebrovascular abnormalities.

Several studies have documented outcomes for patients with Anx/Dep who undergo other types of surgeries. For example, a study of male veterans who had a coronary artery bypass graft found that a history of depression contributed to increased cardiac hospitalizations, continued surgical pain, and decreased likelihood of returning to their previous level of activity [2]. Our study adds to the literature that reports worse QOL outcomes for patients with poor mental health.

Contrary to our hypothesis that patients with Anx/Dep would have worse outcomes at all follow ups, we found that the initial effect of worse GMH scores at 30 day follow up disappeared by six months. A study of self-reported QOL outcomes for the endovascular repair of thoracoabdominal aortic aneurysms also described this in their cohort sample [6]. Using the RAND SF-36, a short form questionnaire that assesses the effects of physical and mental health on overall QOL, researchers found that patients had reduced physical and mental health while in the hospital; however, they returned to their baseline QOL status by six months [6]. Our broader inclusion criteria including age range and procedure type allowed for a more representative and generalizable sample in the setting of cerebrovascular disease. Additionally, cerebrovascular disease affects the brain, and therefore may differ in terms of mental and physical outcomes compared to repair of thoracoabdominal aortic aneurysms.

Our study had some limitations. First our study was retrospective in nature. Second, we relied on self-reported Anx/Dep from the patients’ medical records. Third, the sample lacked diversity in ethnicity. Fourth, patients who were lost to follow up at 30 days may have created a bias in the type of patient that was included in the analysis; however, we were most interested in QOL outcomes which required follow up data from at least one time point. We think it is less likely that we introduced a selection bias given that their baseline GMH scores and likelihood of Anx/Dep was not different between those who were included and those who were excluded from this study.

Our study has several strengths. First, we were able to use detailed sociodemographic and hospital complication data including many possible confounding variables to complete a multivariate analysis to determine QOL outcomes. Second, we focused on cerebrovascular procedures which, to our knowledge, has not been reported in the literature to date, and used widely accepted scales for functionality and overall global health. We also used a wider variety of cerebrovascular disease diagnoses than other studies, and had data available up to six months.

## Conclusion

In summary, our study found that patients with Anx/Dep had worse GMH outcomes at 30 days after their cerebrovascular procedure, while their physical scores were unaffected. Additionally, their GMH scores fell back into the average range by six months after their procedure. This knowledge could be used to better prepare patients who present for an elective procedure who also have Anx/Dep by making hospital resources for mental health available to them prior to and after their procedure. Educating patients with Anx/Dep about their potential for initially worse outcomes may give patients more realistic expectations about what to expect during their recovery. Future research should consider whether patient’s self-reported baseline mental health status is associated with their ability to return to work or their usual daily activities as well as if any mental health interventions pre- or post-procedure would be beneficial.

## Data Availability

The datasets used and/or analyzed during the current study are available from the corresponding author on reasonable request.

## Abbreviations

Anx/Dep: Anxiety/Depression
GHS: Global Health Scale
GMH: Global Mental Health
GPH: Global Physical Health
ICU: Intensive Care Unit
IPH: Intraparenchymal Hemorrhage
mRS: Modified Rankin Scale
QOL: Quality of Life
